# National Institutes of Health R-series Grants portfolio of racism and healthcare, 2017–2022

**DOI:** 10.1186/s12889-023-17407-8

**Published:** 2023-12-14

**Authors:** Judith Aponte, Maria Isabel Roldós

**Affiliations:** 1 Hunter College School of Nursing| City University of New York – CUNY, CUNY Institute of Health Equity, New York, USA; 2 Department of Health Equity, Administration, and Technology, School of Health Sciences, Human Services and Nursing (HS2N) | Lehman College | City University of New York – CUNY, CUNY Institute for Health Equity, New York, USA

**Keywords:** National Institutes of Health, R-series Grants, Racism and healthcare

## Abstract

**Background:**

Any form of racism in healthcare is an unacceptable barrier to receiving equitable and quality care, further contributing to health disparities among populations. For these reasons, it is critical to have a better understanding on the amount of research and scientific advances of funded projects aimed at racism in healthcare. An examination of the distribution of R-series funded research by the National Institutes of Health (NIH) on racism in healthcare during a 5-year fiscal year (FY) period (2017–2022) was conducted by the study team.

**Methods:**

This cross-sectional study used publicly available data from the NIH RePORTER (Research Portfolio Online Reporting Tools: Expenditures and Results) for research project grants awarded on racism and healthcare during the FYs of 2017 to 2022. The number of R-series NIH funded projects on racism in healthcare were examined, including the abstract and public health relevance statement, number of publications, spending category, fiscal start and end dates, total amount of funding each year, funding agency/center(s), and type of funding opportunity announcements. Descriptive statistics were performed on the data by the research team.

**Results:**

There were a total of 93 R-series grants funded during the FYs of 2017 to 2022. Most of the grants were R01s (77.4%); focused primarily on racism at the system-wide level (68.8%), and on patients (64.5%); the largest racial and ethnic minority group reported were African/American/Black (20.4%); and close to 40% did not report race or ethnicity. None of the grants focused in internalized racism, which is at the individual –level. From the FYs of 2017 to 2022, 0.07% of all NIH research funding was awarded to racism in healthcare.

**Conclusion:**

The findings of this study showed the need for continued funding and of the need of more research on racism in healthcare, that potentially can reduce health disparities and inequities.

## Introduction

Racism is a form of discrimination to a specific race or ethnicity [1, 2]. The Office of Management and Budget–defines ethnicity as: Hispanic or not Hispanic and has identified five races (White, Black, American Indian/Alaska Native, Asian, Native Hawaiian/Pacific Islander) [3]. The most extreme effect of racism can result in emotional and mental health trauma, as well as chronic stress [4]. Racism in the health care setting is manifested in distrust of healthcare providers and/or systems, potentially resulting in poor health seeking behaviors and/or decrease use of preventative services [5].

Racism leads to inequities and contribute to health disparities; is an expression of marginalization and oppression, and is based on unfounded beliefs of inferior status [6–8]. Studies have shown populations, such as Black and Hispanic, to have experienced racism in the healthcare setting; to have reported receiving worse care, poorer physical and psychological health outcomes, to have an inadequate patient-provider relationship, and higher morbidity and mortality rates when compared to their White counterparts [8–11]. Further, health outcomes from the COVID-19 pandemic has shown continued racial and ethnic inequity within healthcare, and the need to focus initiatives and resources that address racism [12].

Addressing racism is a complex concept, as it is multidimensional and impacts people at many levels of healthcare. Racism is a root cause of health inequities and are considered upstream factors (i.e., conditions and circumstances that provide the context on the source of the problem that shape the behaviors of individuals) [13]. Within racism, these upstream factors produce downstream effects, consequences and inequities, by affecting individuals and resulting in physical and mental health-related morbidities [14].

## Literature review

The literature recognizes four major types of racism seen in healthcare: 1) internalized 2) interpersonal; 3) institutional; and 4) structural [14]. Although all forms of racism are regarded upstream factors [13], both internalized and interpersonal are forms that are at the individual-level or micro-level; while institutional and structural are system-wide or at the macro-level [14, 15].

*Individual-level.* Individual or micro-level forms of racism are called internalized or interpersonal racism [14]. Internalized racism is within individuals, resulting in conscious and unconscious acceptance of negative stereotypes of the respective racial group [16, 17]. Internalized racism has been seen to affect individuals, at all ages including those as young as 3 years old [18]. Studies have shown internalized racism to cause psychological distress (e.g., depression, anxiety) and result in internalized oppression [16, 19].

Unlike internalized racism which is within an individual, interpersonal is between individuals [19]. Interpersonal racism includes covert (e.g., implicit bias) or overt actions toward others [4]. Within healthcare, internalized racism can negatively impact the patient-provider relationship [20]. Racism in the patient-provider relationship may result in individuals being excluded or in receiving differential health treatments compared to other groups [17, 20]. For example, a United States (US) study reported that healthcare providers denied their African American patients pain medications due to racism, assuming they may misuse them [21]. This has been particularly seen among those with sickle cell disease, where a lack of access and delivery of high-quality care has been denied to African American patients, stemming from interpersonal and/or structural racism [22].

*System-level.* Institutional, systemic or structural racism, is the term used to describe forms of racism that exist in policies or within institutions or organizations; and foster discrimination by reinforcing inequitable macro-level systems that then support and enable discriminatory beliefs and practices [23–25]. These three forms of racism (i.e., institutional, systematic or structural) are embedded in policies, systems, and/or practices that generate unfair treatment and oppression of a specific race resulting in negative outcomes [6]. System level racism has been and remains to be a fundamental driver of health disparities [26].

Associations and causes of racism within healthcare have been emphasized and noted primarily as disparities among different races [20, 27, 28]. For example, a US study reported racial disparities in infant and maternal health and showed Black (3-times higher), and American Indian and Alaska Native (2-times higher) women to have higher rates of pregnancy-related deaths compared to White women [29, 30]. In the US, infant mortality rates are higher among non-Hispanic Black individuals (10.6 deaths per 1000 births) compared to non-Hispanic Native Hawaiian or other Pacific Islander (8.2 deaths per 1000 births), non-Hispanic American Indian or Alaskan Native (7.9 deaths per 1000 births), Hispanic (5 deaths per 1000 births) or non-Hispanic White people (4.5 deaths per 1000 births) [31]. The U.S. Centers for Disease Control and Prevention (CDC) [30] reports that even though many social determinants of health, such as healthcare quality and underlying health conditions contribute to infant and maternal health disparities and inequities, they are still rooted in some form of racism (e.g., implicit bias).

Racism is a major contributor of inequities and for this reason research on racism in healthcare must continue [32]. A way to address racism in healthcare is through the allocation of funds. Funding is one of the major ways to identify opportunities to improve care, and develop and sustain programs [33]. Federal funding through grants allow scientists to seek an understanding of the causes of health disparities in order to advance and improve human health, reduce burden of disease and achieve health equity [34, 35]. One of the largest public funding sources of biomedical research in the US is the National Institutes of Health (NIH). NIH is an important stakeholder in understanding the effects of racism on health. NIH is made up of 27 different institutes and centers, each with their own specific research agenda focused on specific diseases, illnesses, or populations [36]. Although racism has been linked to poor health outcomes, little is known on the type of R-series research, population focus and scientific advances of funded projects aimed at racism in healthcare, and for this reason this portfolio analysis was conducted.

## Methods

### Grant search strategy

Our study team conducted one search of funded NIH grants during January 1 and 9, 2023. Selective text query in the NIH’s Research Portfolio Online Reporting Tools- RePORTER (https://reporter.nih.gov/) was used by the research team [36]. This cross-sectional study used RePORTER, which is a publicly available website that provides access to descriptions of funded NIH grants. Five fiscal years were examined in this NIH portfolio analysis (2017–2022). The analysis includes a vast amount of information, including the title of the grant, project number, abstract text, public health relevance statements, NIH spending category, project start and end date, publications, funding history, and type of funding opportunity announcements (FOAs).

### Statistical analysis

#### Screening and eligibility

Five federal fiscal years (2017–2022) were examined as it represents one RO1 funding cycle. The research team used a three-step procedure of the screening and eligibility of the grants; and are detailed in Table 1.

**Table 1:** Three step screening and eligibility process

First step entailed: 1. Identification of NIH funded grants through a search that used two keywords that were free terms, *racism* and *healthcare*, resulted in a total of 558 funded proposals representing all types of grants (e.g., R-, P-series). 2. Non-R-series grants were removed (278 grants). 3. The remaining 280 abstracts and public health relevance statements were exported into excel and then into Covidence. Although Covidence [37] is a cloud-based collaborative platform designed specifically for the management of reviews, it was used in this study by the research team. 4. After uploading them into Covidence, duplicates were identified and removed; and 90 duplicates were eliminated, resulting in a total of 190 R-series grants.	
Second step included: 1. The inclusion and exclusion criteria were used during the search and screening phase. The inclusion criteria were: 1) research based R-series grants; 2) focused on racism in healthcare; 3) conducted by US-based entities and within the US territories; and 4) conducted during the five fiscal years of 2017 to 2022. The exclusion criteria were grants: 1) without a focus on racism in healthcare; and 2) non-US-based. Given that research-based grants were the emphasis of this study, R-series were the only types of grants focused on in this portfolio analysis. 2. Both the inclusion and exclusion criteria were entered into Covidence and used independently by each of the two reviewers to appraise the 190 R-series abstracts and public health relevance statements. Each reviewer took notes in the platform and answered the following question: Does the purpose/aim state that the study plans to address racism among healthcare users and within healthcare? 3. After the independent reviews were completed, the first author saw the recommendations (those with consensus or disagreement) in Covidence, and for any discrepancies, the reviewers met via ZOOM to discuss and resolve them by consensus; and 97 were excluded for not meeting the inclusion criteria. The reviewers had a 90% agreement rate.	
During the third step: 1. Data were extracted from the remaining 93 abstracts and public health relevance statements. 2. In preparation for data extraction, the quality assessment template in Covidence was used to rate for risk of bias. The data extraction template feature in Covidence was also used by each of the two reviewers, but they met via ZOOM to discuss and customize the headings in Table 3. 3. Other data extracted and analyzed from the NIH RePORTER project sections was the NIH Research, Condition, and Disease Categorization (RCDC) spending categories, number of publications from each corresponding grant, funding history and funding opportunity announcements, during the fiscal years 2017 to 2022 (Table 3).	

**Fig. 1:**
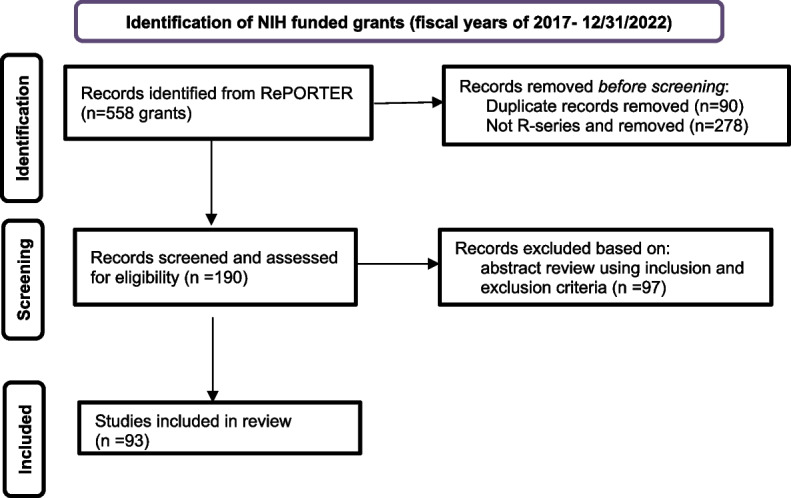
PRISMA 2020 flow diagram: Racism and healthcare

The results of the search are outlined in the PRISMA flow diagram (Fig. 1) [38]. The PRISMA flow diagram shows the flow of information through the various stages of the screening and eligibility review; as it outlines the number of records identified, included and excluded, the reasons for exclusions, and the final number of studies reviewed [39].

Descriptive statistics were calculated. Given that the study did not involve human subjects, institutional review board approval was not needed. For this study the research team used the Strengthening the Reporting of Observational Studies in Epidemiology (STROBE) reporting guideline checklist [40].

## Results

Results from this portfolio analysis showed that during the FYs of 2017 to 2022 NIH funded 93 grants on racism in healthcare (see Table 2 and Table 3). The largest R-series mechanism funded were R01s (77.4%), and most FOAs were program announcements (PAs) or program announcement receipt (PAR-a type of PA; 64.5%). More than half (68.8%) of the studies focused solely on racism at the system-wide level (i.e., structural or institutional), while few (16.1%) were on the individual-level (i.e., internalized and interpersonal racism), or included both forms of racism (15.1%; i.e., individual and system-wide; see Table 2 and Table 3). Based on this, most publications (57.9%) disseminating the findings of the studies focused on system-wide racism in healthcare. (Table 2).

**Table 2:** Description of grants funded from 2017 to 2022: Type, length, population and number of publications

Type of Grant	No. of grants *n* = 93 (%)	No. of publications *n* = 278 (%)
Type of racism		
*System-wide*	64 (68.8%)	161 (57.9%)
Structural or systemic	61 (66%)	145 (52.2%)
Institutional	1 (1.1%)	16 (5.7%)
Structural or systemic and institutional	2 (2.15%)	0 (0%)
*Individual-wide*	15 (16.1%)	82 (29.5%)
Interpersonal	15 (16.1%)	82 (29.5%)
Internalized	0 (0%)	0 (0%)
*Both system- and individual-wide*	14 (15.1%)	35 (12.6%)
R-series Type		
R01	72 (77.4%)	259 (93.2%)
RF1	2 (2.15%)	0 (0%)
R03	1 (1.1%)	0 (0%)
R13	2 (2.15%)	0 (0%)
R21	8 (8.6%)	16 (5.7%)
R25	4 (4.3%)	2 (0.7%)
R34	1 (1.1%)	1 (0.4%)
R36	3 (3.2%)	0 (0%)
Grant length and number of publications per category		
< 1–1 year	5 (5.4%)	18 (6.5%)
2–4 years	27 (29%)	81 (29.1%)
5 years and longer	61 (65.6%)	179 (64.4%)
Type of FOA		
PA or PAR	60 (64.5%)	232 (83.5%)
RFA	33 (35.5%)	46 (16.5%)
Health disparity populations		
Racial and ethnic minority only (Black, Hispanic, Asian, American Indian/Alaska Native, Native Hawaiian/Pacific Islander), including “people of color”	44 (47.3%)	87 (31.3%)
African American/Black only	19 (20.4%)	26 (9.4%)
Hispanics/Latinos/Latinas/Latinx only	0 (0%)	0 (0%)
Asians only	2 (2.15%)	0 (0%)
“People of color” only	3 (3.2%)	0 (0%)
African American/Black and Hispanics only	12 (12.9%)	104 (37.4%)
American Indian/Indigenous only	1 (1.1%)	0 (0%)
Asians and mixed ethnicity/race included	1 (1.1%)	1 (0.4%)
Ethnicity/race not reported (NR)	37 (39.8%)	120 (43.2%)
More than one racial and ethnic minority population	22 (23.7%)	118 (42.4%)
Funding dollar amounts: NIH Research	Projects on racism in health care (*n* = 93)	Overall research projects (*n* = 232,482)
	$91,889,931 (0.07%)	$130,105,310,674

^a^ Of 93 grants there were 173 funding amounts

**Table 3:** NIH RePORT R-series Funded Grants on Racism in Health Care from Fiscal Year (FY) 2017–2022

No.	Series/ FOA	Title	Population (race/ethnicity)	Focus	Type of racism	Project Start-End	FY (dollar amount)	Funding Agency
1	R01/ PAR	Accountability for Cancer Care through Undoing Racism and Equity (ACCURE)	Breast & lung cancer (African American)	Patient (pt) & providers	Institutional	06/01/12–03/31/18	2017; ($149,065)	National Cancer Institute (NCI)
2	R01/ RFA	Addressing Structural Disparities for Children with Early Communication Disorders (ASCEND)	Children with early communication disorders (people of color)	pt	structural racism (SR)	06/01/22–05/31/27	2022; ($717,005)	National Institute on deafness and other communication disorders (NIDCD)
3	R25/ PA	Addressing vaccine hesitancy in Baltimore City through a youth engagement/ health literacy STEM initiative	High school students (NR)	pt	SR	09/01/18–06/30/23	2021; ($54,000)	National Institute of General Medical Science (NIGMS)
4	R01/ PA	Advancing knowledge on factors that promote or impede engagement along the HIV care continuum over time: A longitudinal mixed methods study of Black and Latinx youth/ emerging adults living with HIV	Young people living with HIV (African American/ Black or Latinx)	pt	interpersonal racism (IR)	07/01/21–05/31/25	2021; ($761,164) 2022; ($726,342)	National Institute on Drug Abuse (NIDA) NIDA
5	R01/ PA	Advancing understanding of racism-related health disparities beginning before birth: A multisite study with Black and Latina pregnant women	Pregnant women (Black and Latina)	pt	IR	09/07/22–06/30/27	2022; ($698,116)	Eunice Kennedy Shriver National Institute of Child Health & Human Development (NICHD)
6	R01/ PA	African American Resilience in Surviving Cancer	Cancer survivors (African American)	pt	SR	09/12/19–08/31/24	2019; ($623,694) 2020; ($623,695) 2021; ($623,694) 2022; ($611,223)	NCI NCI NCI NCI
7	R25/ PA	Aging in the time of COVID: Racism, Isolation, and Meaning	Adults-65 years and older (Black, Indigenous and peoples of color; BIPOC)	pt, providers & students	systemic racism (SYR) & IR	05/01/19–02/29/24	2021; ($107,643)	NIH Office of the Director (OD)
8	R01/ PA	An internet-based preconception cohort study in North America	Preconception cohort (NR)	pt	IR	09/14/16–04/30/27	2022; ($708,812)	NICHD
9	R01/ PA	A Non-Inferiority Trial Testing Delivery of Written Exposure Therapy by Community Health Workers	Pregnant women with PTSD (NR)	pt & providers	IR	08/17/22–07/31/27	2022; ($562,952)	NICHD
10	R01/ RFA	Applying Critical Race Theory to investigate the impact of COVID-19-related policy changes on racial/ethnic disparities in medication treatment for opioid use disorder	Opioid use disorder (Black, Hispanic/ Latinx, & non-Hispanic Whites; NHWs)	pt	SR	04/01/22–01/31/27	2022; ($662,421)	NIDA
11	R01/ RFA	A Pragmatic Trial of Integrating Community-based Patient Navigation into the Continuum of Maternal Care for Black Women in a Safety-Net Health System: Effects on Maternal Health, Health Care, Morbidity	Maternal care (Black)	pt	SYR	09/23/22–06/ 30/27	2022; ($714,617)	National Institute of Nursing Research (NINR)
12	R21/ PA	A Sequential Mixed Methods Study Evaluating the Influence of Violence on HIV Care and Viral Suppression among Young Black and Latinx MSM	Men that have sex with men (MSM) (Black & Latinx)	pt	IR	07/01/20–05/30/23	2021; ($275,917) 2021; ($214,533)	National Institute of Mental Health (NIMH) NIMH
13	R03/ PA	Automated Substance Use Detection from Electronic Health Records in the Pediatric Setting	Adolescents (NR)	pt	SYR	04/01/22–03//31/24	2022; ($79,500)	NIDA
14	R01/ PA	Buffering effects of a tiered preventive model on parent adjustment, parent-child relational health, and child psychosocial development post COVID-19	Low-income families (Black & Latinx)	pt	IR	06/01/14–06/30/23	2021; ($162,069) 2022; ($162,137)	OD NICHD
15	R01/ PA	Cervical Researchers Empowerment Women: Engagement for Multi-Level Intervention	Cancer prevention & care (NR)	pt	SR	12/01/18–11/30/23	2021; ($24,130)	NCI
16	R25/ PAR	Clinical Research Scholars Training (CREST) Program	Training (NR)	providers	SR	07/01/22–01/28/27	2022; ($528,500)	National Institute on Minority Health and Health Disparities (NIMHD)
17	R01/ PA	Contextual Determinants of Sexual Minority Health in the United States	Sexual minority health (NR)	general population (pop)	SR	07/21/22–03/31/25	2022; ($350,192)	NIMHD
18	R21/ PA	Cultural Consensus Modeling to Identify Culturally Relevant Risk Factors for Suicide among Black Youth	Youth (Black)	pt	SR	08/15/22–07/31/24	2022; ($265,259)	NIMH
19	R34/ PA	Developing a Data-Driven Management System Using Machine Learning and Mixed-Methods Research to Predict Job Turnover Among Mental Health Professionals	Employees (Black)	providers	SR	12/18/19–11/30/222	2020; ($244,635) 2021; ($38,768) 2021; ($230,630) 2022; ($107,000) 2022; ($223,506)	NIMH NIMH NIMH NIMH NIMH
20	R01/ PA	Development of a Risk Factor Screen for Mental Health Problems after Sudden Illness or Injury	Mental health (African American, Latino, Asians, & mixed ethnicity/race)	pt	IR	09/20/17–04/30/21	2017; ($693,637) 2018; ($626,313) 2019; ($636,599) 2020; ($610,092)	NIMHD NIMHD NIMHD NIMHD
21	R01/ RFA	DiSRUPT: Dismantling Structural Racism Underlying the Organization of Ambulatory PracTices: an observational study of clinical desegregation	Medicaid population (Black & Latinx)	pt & providers	SR, IR & SYR	06/19/22–02/28/27	2022 ($844,995)	NIMHD
22	R13/ PA	Diversity, Equity and Inclusion: Developing a research agenda for addressing Racism in Emergency Medicine	Conference (NR)	providers	IR & SR	01/01/22–12/31/22	2022; ($50,000)	Agency for Healthcare Research and Quality (AHRQ)
23	R01/ PA	Diversity Supplement: Implementation of EMR-Integrated Referrals	Adults 18–64 years (Black)	pt	SR	01/01/20–12/31/24	2022; ($91,857)	National Heart, Lung, and Blood Institute (NHLBI)
24	R21/ PAR	EleVATE-Clinicians: a tool to mitigate implicit bias by increasing clinicians’ empathy	Pregnant women (NR)	pt	SR	09/23/22–06/30/24	2022; ($217,298)	NIMHD
25	R01/ RFA	Ending the HIV Epidemic with Equity: An All-facility Intervention to Reduce Structural Racism and Discrimination and Its Impact on Patient and Healthcare Staff Wellbeing	Living with HIV (BIPOC)	pt & providers	SR	07/01/22–04/30/27	2022; ($848,121)	NINR
26	R01/ PAR	Enhancing Perinatal Care Support to Improve Maternal Mortality Disparities	Pregnant women (BIPOC)	pt	IR & SYR	08/24/21–04/30/26	2021; ($622,309) 2022; ($581,977)	NIMHD NIMHD
27	R21/ PA	Evaluation and validation of a novel instrument to assess the psychosocial and drug history backgrounds of pregnant women with or without Opioid Use Disorder	Pregnant women (NR)	pt	SR	09/30/20–08/31/23	2020; ($197,694) 2021; ($47,403 & $384,948, respectively) 2021; ($237,569)	NIDA NIDA OD NIDA
28	R01/ RFA	Examining the Impact, Pathways, and Cost of County-Level Structural Racism on Hypertension Disparities in Black and White US Adults	Hypertension (Black & White)	pt	SR	07/20/22–05/31/26	2022; ($453,372)	NHLBI
29	RF1/ RFA	Examining racial segregation and underlying mechanisms related to VCID and incident stroke in the REGARDS study	Dementia, cognitive impairment & stroke (NR)	pt	SR	05/01/22–04/30/25	2022; ($2,258,218)	National Institute on Aging (NIA)
30	R01/ RFA	Healthcare Organizational Structural Conditions and the Health of People Recently Released from Prison	Formally incarcerate individuals (BIPOC)	pt & policy	SR	09/07/22–06//30/27	2022; ($790,519)	NHLBI
31	R01/ PA	Health System and Contextual Factors Associated with Racial Equity in Lung Cancer Care	Medicare (non-Latinx Black & non-Latinx White)	pt & providers	SR	09/21/22–05/31/27	2022; ($629,894)	NIMHD
32	R01/ RFA	Hospital quality, Medicaid expansion, and racial/ethnic disparities in maternal mortality and morbidity	Medicaid (NR)	pt	SR	09/17/20–06/30/25	2020; ($699,107) 2021; ($624,345) 2022; ($614,019)	NIMHD NIMHD NIMHD
33	R01/ PA	Housing assistance, Outcomes, Medicare, and SEER (HOMES): using a novel data linkage to understand cancer inequities	Cancer care & Medicare (NR)	pt	SYR	07/01/22–06/30/27	2022; ($581,773)	NCI
34	R01/ RFA	Impact of Structural Racism and Discrimination on Liver Disease Disparities in High-Risk Asian American Populations	Liver disease (Asian Americans)	pt	SR	09/15/22–05/31/27	2022; ($838,349)	NIMHD
35	R01/ RFA	Improving Chronic Disease Outcomes Across the Lifespan by Addressing Structural Racism	Chronic disease (NR)	pt & providers	IR & SR	09/23/22–06/30/27	2022; ($889,418)	NINR
36	R01/ PA	Improving Health Outcomes and Equity by Targeting Postpartum Mothers at Highest Risk	Postpartum moms (Black & Latina)	pt	IR	09/18/20–05/31/26	2020; ($634,448) 2021; ($1,274,899) 2021; ($709,546)	NIMHD OD NIMHD
37	R01/ PA	Improving Pediatric Brain Injury Outcomes Through Equitable Care Implementation	Pediatric brain injury (NR)	pt	SYR	07/01/22–06/30/23	2022; ($100,000)	National Institute of Neurological Disorders and Stroke (NINDS)
38	R01/ PA	Improving racial equity in opioid use disorder treatment in Medicaid	Opioid use disorder (NR)	pt & providers	SR	09/01/22–06/30/27	2022; ($1,576,154)	NIDA
39	R01/ PAR	Improving the Collaborative Health of Minority COVID-19 Survivor & Carepartner Dyads Through Interventions Targeting Social and Structural Health Inequities	COVID-19 survivor (African American)	pt & policy	SR	09/15/21–06/30/26	2021; ($436,141 & $250,000; respectively) 2022; (679,180)	OD NINR NINR
40	R01/ RFA	Improving the Organizational Social Context to Address Structural Racism and Discrimination: A Randomized Controlled Trial to Reduce Racial Disparities in Viral Suppression and Retention in HIV Care	HIV care (NR)	pt	SR	09/16/22–06/30/27	2022; ($817,490)	NINR
41	R01/ RFA	Increasing engagement and improving HIV care outcomes via stigma reduction in an online social networking intervention among racially diverse young men who have sex with men and transgender women	Young Black or Latino men who have sex with men & transgender women (YBLMT) (Black & Latino)	pt	SR	08/16/18–03/31/23	2018; ($816,583) 2019; ($764,780) 2020; ($715,387) 2020; ($100,152) 2021; ($719,097) 2021; ($100,949) 2022; ($720,835)	NIMHD NIMHD NIMHD NIMHD NIMHD NIMHD NIMHD
42	R01/ RFA	Investigating structural maternal health inequities among Black reproductive aged women in Georgia: a mixed methods and multi-level approach	Maternal health (Black & White)	pt & providers	institutional & SR	09/19/22–05/31/27	2022; ($302,441 & 150,000, respectively)	NICHD OD
43	R01/ PA	Leveraging Community-Academic Partnerships and Social Networks to Disseminate Vaccine-Related Information and Increase Vaccine Uptake Among Black Individuals with Rheumatic Diseases	Rheumatic disease (Black)	providers	SR & SYR	05/10/22–04/30/27	2022; ($791,024)	National Institute of Arthritis and Musculoskeletal and Skin Disease (NIAMS)
44	R01/ RFA	Mitigating Structural Racism to Reduce Inequities in Sepsis Outcomes	Sepsis (NR)	providers	SR	04/01/22–03/31/27	2022; ($552,246)	NIGMS
45	Rf1act/ RFA	Moving Upstream: Understanding Racism, Firearm Injury Risks, and Resiliency Among Asian Americans	Firearm injury risk (Asian American)	pt	IR & SR	09/15/22–06/30/25	2022; ($2,162,049)	OD
46	R01/ PA	Multilevel Determinants of Racial and Ethnic Disparities in Maternal Morbidity and Mortality in the Context of COVID-19 Pandemic	Postpartum women (Black & Hispanics)	pt & providers	SR	09/07/21–05/31/23	2021; ($886,186)	OD
47	R01/ RFA	Multilevel Forms of Structural Racism and Racial Inequalities in ADRD Risk	Alzheimer’s Disease Related Dementias (ADRDs) (Black & White)	pt	SR	07/01/22–03/31/27	2022; ($720,008)	NIA
48	R21/ RFA	Multilevel Racism & Discrimination and PrEP Outcomes Among Black SMM in the Southeastern U.S.	Sexually minority men (SMS) (Black)	pt	SR	09/08/22–06/30/27	2022; $277,306 & $341,393, respectively)	NIMH OD
49	R01/ PA	Neighborhoods, Networks, and the HIV Care Continuum among HIV-infected MSM in NYC	MSM (Black)	pt	IR	05/01/19–02/29/24	2019; ($843,949) 2020; ($859,867) 2021; ($834,807) 2022; ($212,918) 2022; ($787,974)	NIMH NIMH NIMH NIMH NIMH
50	R01/ PA	Offering Women with PrEP with Education, shared decision-making and trauma-informed care: the OPENS trial	HIV prevention (Black)	pt	institutional & SYR	09/01/18–03/31/23	2018; ($530,692) 2019; ($495,571) 2019; ($262,307) 2020; ($469,972) 2021 ($79,087) 2021 ($484,726) 2022 ($474,791) 2022 ($116,270)	NIMHD NIMHD OD NIMHD NIMHD NIMHD NIMHD NIMHD
51	R01/ RFA	Pathways to Reducing Disparities in Depression Outcomes	Homeless men & women (African Americans & Latinos)	pt	IR, SR and SYR	07/10/14–03/31/19	2017 ($416,161)	NIMHD
52	R01/ PAR	Perceived racism, cardiovascular disease risk, and neurocognitive aging	Cardiovascular risk & seniors (Black)	pt	Institutional SR & IR	08/01/22–04/30/27	2022; ($831,723)	NIA
53	R01/ PA	Placental origins of phthalate-induced changes in fetal reproductive development	Maternal health (Black & White)	pt	SR	09/13/21–04/30/22	2018; ($661,408) 2019; ($588,674) 2020; ($570,795) 2021; ($538,933) 2022; ($29,057)	National Institute of Environmental Health Sciences (NIEHS) NIEHS NIEHS NIEHS NIEHS
54	R01/ PA	Pregnancy-Associated Mortality	Pregnancy (NR)	pt	SR	08/27/18–06/30/23	2018; ($263,375) 2019; ($265,125) 2020; ($303,193) 2020; ($242,616) 2021; ($284,388) 2022; ($284,388)	NICHD NICHD NICHD NICHD NICHD NICHD
55	R01/ PA	Prenatal to Preschool: The Impact of the Pandemic on Mothers and children, with a focus on syndemic effects on Black families	Pandemic & mothers & children (Black & non-Latinx White)	pt	IR & SR	07/06/22–05/31/27	2022; ($834,125)	NIMH
56	R01/ RFA	Promoting alcohol treatment engagement post-hospitalization with brief intervention, medications, and CBT4CBT: A randomized clinical trial in a diverse patient population	Alcohol treatment (NR)	pt	SR	09/20/21–05/31/26	2021; ($670,645) 2022; ($727,945)	National Institute on Alcohol Abuse and Alcoholism (NIAAA) NIAAA
57	R01/ PA	Promoting children’s oral health: Identifying provider-, practice-, and community-level characteristics associated with delivery of fluoride varnish in medical offices	Children’s oral health (NR)	pt & providers	SR	09/15/22–05/31/23	2020; ($430,890) 2021; ($456,806) 2022; ($416,857) 2022; ($426,468)	OD National Institute of Dental & Craniofacial Research (NIDCR) OD NIDCR
58	R01/ PA	Psychosocial stress, cardio-respiratory fitness, and the medial temporal hippocampal system in Black emerging adults	Psychosocial stress (Black)	pt	IR & SR	08/05/22–05/31/27	2022; ($871,991)	NIMH
59	R01/ PA	Racial disparities in preterm births and fetal losses	Preterm births (non-Hispanic Blacks (NHBs) & NHWs)	pt	SR	08/15/21–06/30/26	2021; ($587,003) 2022; ($504,355) 2022; ($74,520)	NICHD NICHD NICHD
60	R01/ PA	Racial/Ethnic Disparities in Ovarian Cancer Treatment and Survival: An Integrative Approach	Ovarian cancer (African American, Hispanic, Asian, Pacific Islander, & NHW)	pt	SR	07/01/20–06/30/25	2020; ($1,371,104) 2021; ($1,386,624) 2022; ($1,445,236) 2022; ($102,222) 2022; ($199,995)	NCI NCI NCI NCI NCI
61	R01/ PA	Racism, Residential Racial Segregation, and Breast Cancer Survival Disparities among Black, Hispanic and non-Hispanic White Women	Breast cancer survival (Black, Hispanic & NHW)	pt & policy	SR	05/08/17–04/30/22	2017; ($462,342) 2018; ($455,960) 2019; ($451,945) 2020; ($475,937) 2020; ($62,161) 2021; ($558,055)	NCI NCI NCI NCI NCI NCI
62	R01/ PAR	Randomized Controlled Trial of Indigenous Recovery Planning for American Indians	Substance abuse disorder American Indians (AIs)	pt	SR & SYR	04/01/21–02/28/26	2021; ($663,688) 2022; ($630,504)	NIDA NIDA
63	R01/ RFA	Research Employing Environmental Systems and Occupational Health Policy Analyses to Interrupt the Impact of Structural Racism on Agricultural Workers and Their Respiratory Health (RESPIRAR)	Migrant & seasonal farmworkers & respiratory health (Black &/or Latinx)	pt & policy	SR	09/01/22–06/30/27	2022; ($767,058)	NIEHS
64	R13/ PA	Society of Behavioral Medicine 2022 Annual Meeting & Scientific Sessions	Scientific meeting (NR)	providers	SYR	04/25/22–03/31/23	2022; ($35,000 & $30,000, respectively) 2022; ($4087)	NHLBI OD OD
65	R01/ PA	State-level factors and maternal and child health outcomes	Maternal & child health (NR)	pt & policy	SR	09/30/22–06/30/27	2022; ($439,100)	NICHD
66	R01/ PA	Structural and Social Determinants of Maternal Mental Health, Morbidity, and Inequities in COMBO	Maternal & mental health (NR)	pt & providers	SR	05/01/21–03/31/24	2021; ($881,466)	NIMH
67	R01/ RFA	Structural Influences on Methamphetamine Use among Black Gay and Bisexual Men in Atlanta	Gay & bisexual men (Black)	pt	SR	06/01/22–03/31/27	2022; ($724,152)	NIDA
68	R01/ PA	Structural racism and cardiovascular disease risk in pregnant women and their infants	Cardiovascular disease & pregnant women (NR)	pt	SR	12/07/22–07/31/25	2022; NR	NHLBI
69	R01/ RFA	Structural Racism and Discrimination in Emergency Department Transfers: Unintended Consequences of the Emergency Medical Treatment and Labor Act (EMTALA)	Emergency department length of stay (minority & NHW)	pt	SR	05/01/22–12/31/26	2022; ($714,624)	NIMHD
70	R01/ RFA	Structural Racism and Discrimination in the Expansion of Hospital Stroke Care Capacity: A Multi-Level Analysis on Access to Care, Treatment, and Outcomes	Stroke care (NR)	pt	SR	05/14/22–01/31/26	2022; ($562,691)	NIMHD
71	R01/ RFA	Structural Racism and Engagement of Family Caregivers in Serious Illness Care	Serious illness care (NR)	pt & policy	SR & SYR	04/15/22–01/31/27	2022; ($780,733)	NINR
72	R01/ RFA	Structural racism in schools: Evaluating the impact of academic tracking and de-tracking on substance use and health during adolescence and the transition to adulthood	9th to 12th graders (NR)	general pop	SR	08/01/22–05/31/27	2022; ($701,641)	NIDA
73	R01/ RFA	Targeted Investment and Meaningful Engagement to Improve MCH Outcomes and Rectify Historical Structural Racism: The TIME Study	Infant & maternal child health (Black)	pt	SR	05/15/22–03/31/27	2022 ($525,812 & $100,000, respectively)	NICHD NINR
74	R01/ PA	The Affordable Care Act’s Role in Aging Disparities Before and After Medicare Eligibility	Low socio-economic status (NR)	general pop.	SR	08/15/22–04/30/26	2022; ($511,867)	NIA
75	R25/ RFA	The Agile Nudge University Program	ADRD (NR)	students, fellows, & faculty	IR	08/15/22–04/30/27	2022; ($150,120)	NIA
76	R01/ PAR	The Bariatric Experience Long Term (BELONG) II for Racial and Ethnic Minority Patients	Bariatric (NR)	pt & policy	IR	09/23/19–06/30/23	2019; ($749,290) 2020; ($711,095) 2021; ($703,773) 2022; ($687,277)	NIMHD NIMHD NIMHD NIMHD
77	R01/ RFA	The Impact of Racism on Trajectories of Substance Use, Mental Health and Legal System Contact from Adolescence to Young Adulthood	adolescence to young adulthood & substance use, mental health (NR)	pt	SR	05/15/22–02/28/27	2022; ($765,488)	NIDA
78	R01/ RFA	The Impact of Structural Racism on Racial/Ethnic Disparities in End-Stage Kidney Disease from Healthy Population to Mortality	End-stage renal disease & Medicare (NHB, Hispanic, AI or Alaska Native (AN)	pt	SR	08/01/22–05/31/26	2022; ($756,925)	National Institute of Diabetes and Digestive and Kidney Diseases (NIDDK)
79	R01/ RFA	The Influence of Structural Racism on Incidence of Alzheimer’s Disease and Related Dementias (ADRD) in Black women	ADRD & women (Black)	pt	SR	06/01/22–02/28/27	2022; ($799,898)	NIA
80	R36 PA	The Intersecting Effect of Substance Use Stigma, Methadone Treatment Stigma, and Racial Discrimination on Methadone Treatment Outcomes	Substance use & methadone treatment (Black/African American)	pt	IR	08/01/22–07/31/24	2022; ($53,181)	NIDA
81	R21/ PA	The NAME Project: A Narrative Dentistry and Medical Education Project	Dentistry & medical program (NR)	providers & students	SYR	09/01/22–08/31/24	2022; ($281,506)	NIDCR
82	R21/ PA	The Racial Social Structure and Unequal Risk of Adverse Birth Outcomes among Black Infants	Birth & women (Black)	pt	IR & SR	05/05/20–01/31/22	2020; ($241,323) 2021; ($195,824)	NIMHD NIMHD
83	R01/ PA	The Role of Implicit Bias on Outcomes of Patients with Advanced Solid Cancers	Advanced solid cancer (Black & Hispanic)	pt & providers	IR	04/05/21–03/ 31/26	2021; ($708,475) 2022; ($681,760)	NCI NCI
84	R01/ RFA	Transforming Health Equity Research in Integrated Primary Care: Antiracism as a Disruptive Innovation	Systems map (NR)	pt & policy	IR & SYR	08/23/21–04/30/26	2021; ($627,706) 2022; ($617,346)	OD OD
85	R21/ PA	Understanding the COVID-19, Racism, and Violence Syndemic and its Effects on COVID-19 Testing Disparities	COVID-19 (Black)	pt	SYR	05/01/20–06/30/22	2021; ($470,715) 2022; ($441,240)	OD OD
86	R01/ PA	Understanding the impact of racism—a social determinant of health—on the scope of the co-occurrence of mental health and cardiometabolic challenges in high-disparity racial/ethnic minority populations	Mental health (NR)	pt & provider	SR	08/23/21–04/30/26	2021; NR 2022: NR	NIMHD
87	R01/ PA	Understanding the impact of structural racism on racial/ethnic inequities in mortality: The Multiethnic Cohort Study	Mortality (Black, Hispanic/ Latino, Japanese American, & Native Hawaiian)	pt	SR	09/24/22–06/30/27	2022; ($567,735)	NIMHD
88	R01/ PA	Understanding the role of structural racism on racial/ethnic inequities in lung cancer risk	Cancer risk (NR)	pt	SR	07/01/22–06/30/27	2022; ($714,588)	NCI
89	R01/ PA	Universal strengths-based parenting support in pediatric health care for families with very young children following the Flint Water Crisis	Pediatric health (NR)	pt	IR & SYR	03/01/19–02/29/24	2019; ($717,853) 2020; ($671,019) 2021; ($177,211) 2021; ($618,033) 2022; ($160,387 & $605,854, respectively)	NICHD NICHD OD NICHD NIEHS NICHD
90	R01/ PA	Unmet Needs: Achieving Equity and Support for Parents Pursuing Prenatal Diagnosis in the Genomic Era	Perinatal care (Black & Latin-X)	pt	IR	08/12/21–06/30/26	2022; ($155,500)	OD
91	R01/ RFA	Using Disadvantage Indices to Address Structural Racism and Discrimination in Pandemic Vaccine Allocation and Beyond: Defining the Shape of a Novel Paradigm to Promote Health Equity	COVID-19 vaccine (People of color)	pt	SR	07/01/22–06/30/27	2022; ($721,171)	National Institute of Allergy and Infectious Diseases (NIAID)
92	R36/ PAR	Weathering the Storm of Cognitive Inequities: Testing the Minority Stress and Cognition Model with Indigenous Older Adults	Older adults (Black, Indigenous, Hispanic & White)	pt	IR & SYR	07/15/22–06/03/24	2022; ($67,781)	NIA
93	R36/ PAR	Youth in Emergency: how and why youth of color use psychological stabilization services.	Youth & psychological services (people of color)	pt	IR	07/01/21–06/30/23	2021; ($41,516)	NIMH

### Financial and budget analyses

During the FYs of 2017 to 2022, NIH invested a total of $130,150,310,674 in research projects, and yet during this same period $91,889,931 was allocated to the 93 grants on racism in healthcare, representing 0.07% of the total (Table 2). Of the 27 funding agencies/centers, 19 provided funding during these five FYs (2017–2022). Among these 19 NIH agencies/centers, there were 173 funding amounts with multiple agencies/agencies allocating a sum of money during the same year. For example, one 5-year grant had eight amounts of money allocated, which included supplements. Of the 93 grants, there were two that were funded beginning in 2021 and 2022, but under “History,” there were no monies noted for 2021 or 2022 and for this reason no dollar amounts were reported (see Table 3).

The top five NIH agencies/centers that funded the most projects were the National Institute on Minority Health and Health Disparities (NIMHD; 23.7%; 41/173), the NCI (12.1%; 21/173), Eunice Kennedy Shriver National Institute of Child Health Human Development (NICHD; 11.6%; 20/173), the NIH Office of the Director (11%; 19/173), National Institute of Mental Health (NIMH;10.4%; 18/173).

Of NIH’s 315 RCDC spending categories, most of the 93 grants (95.7%; 89/93) reported them; and a total of 127 RCDC spending categories were noted, with the top five categories being, Behavioral and Social Science (88.1%; 82/93); Clinical Research (88.1%; 82/93); Social Determinants of Health and Minority Health (87.1%; 81/93); Minority Health (83.9%; 78/93); and Health Disparities (76.3%; 71/93; Table 4).

**Table 4:** NIH Research, Condition, and Disease Categorization (RCDC) spending category from RePORTER: Grants 2017–2022 (*n* = 127 terms)^a^

Acquired Cognitive Impairment; Adolescent Sexual Activity; Aging; Alcoholism; Alcohol Use and Health; Alzheimer’s Disease Related Dementias (ADRD); Alzheimer’s Disease including Alzheimer’s Disease Related Dementias (AD/ADRD); American Indian or Alaska Native; Anxiety Disorders; Arthritis; Autoimmune Disease; Basic Behavioral and Social Science; Behavioral and Social Science; Behavioral and Social Science Research; Biodefense; Biotechnology; Brain Disorders; Breast Cancer; Cancer; Cardiovascular; Caregiving Research; Cerebrovascular; Cervical Cancer; Childhood Injury; Childhood Obesity; Chronic Liver Disease and Cirrhosis; Chronic Pain; Clinical Research; Clinical Trials and Supportive Activities; Comparative; Effectiveness Research; Conditions Affecting the Embryonic and Fetal Periods; Contraception/Reproduction; Coronaviruses; Coronaviruses Disparities and At-Risk Populations; Coronaviruses Vaccines; Comparative Effectiveness Research; Cost Effectiveness Research; Dementia; Dental/Oral and Craniofacial Disease; Depression; Diabetes; Diagnostics and Prognostics; Digestive Diseases; Dissemination and Implementation Research; Drug Abuse (NIDA only); Emergency Care; Emerging Infectious Diseases; Endocrine Disruptors; Estrogen; Firearms Research; Genetics; Genetic Testings; Health Disparities; HIV/AIDS; Health Effects of Indoor Air Pollution; Health Genome; Health Services; Heart Disease; Hematology; Hepatitis; Hepatitis -B; Homelessness; Homicide and Legal Interventions; Hypertension; Immunization; Infectious Diseases; Infertility; Infant Mortality; Injury (total) Accidents/Adverse Effects; Immunization Infectious Diseases; Kidney Disease; Lead Poisoning; Liver Disease; Lung; Lung Cancer; Lupus; Machine Learning and Artificial Intelligence; Maternal Health; Maternal Morbidity and Mortality; Mental Health; Mental Illness; Methamphetamine; Minority Health; Networking and Information Technology R&D (NITRD); Neurodegenerative; Neurosciences; Nutrition; Obesity; Ovarian Cancer; Opioid Misuse and Addiction; Opioids; Ovarian Cancer; Opioid Misuse and Addiction; Opioids; Pain Research; Pediatric; Pediatric Prevention; Perinatal Period-Conditions Originating in Perinatal Period; Physical Injury - Accidents and Adverse Effects; Post-Traumatic Stress Disorder (PTSD); Pregnancy; Prevention; Preterm, Low Birth Weight and Health of the Newborn; Rehabilitaion; Rural Health; Sexual and Gender Minorities (SGM/LGBT*); Sleep Research; Social Determinants of Health; Stroke; Substance Misuse; Substance Misuse Prevention; Telehealth; Tobacco; Tobacco Smoke and Health; Transplantation; Traumatic Brain Injury (TMI); Traumatic Head and Spine Injury; Unintentional Childhood Injury; Vaccine Related; Vascular Cognitive Impairment/Dementia; Violence; Violence Against Women; Violence Research; Women’s Health	

^a^ The research categories are not mutually exclusive. Some research projects have multiple categories

In order to better understand the amount of monies allocated to racism in healthcare, the research team conducted a sub-analysis of the parent portfolio (93 grants), using a case study that only centered on FY 2022 (Tables 5 & 6).

**Table 5:** Case study: NIH funded research on racism in healthcare for FY 2022

**Case study objective:** This is a sub-analysis to the parent portfolio analysis aimed to determine the level of NIH investment of FY 2022 on racism in healthcare. **Design:** NIH-funded research (R-series) data was queried from the NIH RePORTER system. Extramural research on racism in healthcare, conducted in the United States during the FYs of 2017 to 2022 were examined. Only NIH R-series grants with the FY 2022 funding period, that were on racism in healthcare was the focus of this sub-analysis. **Main Outcomes and Measures:** The grants within FY 2022 were counted, each finding agency/center was identified and the total funding dollars of each of these grants were identified. The funding proportion was calculated using one denominator, which was the NIH expenditures of all R-series funded projects. For all projects during the FY of 2022, NIH funded 38,426 R-series projects with a total investment of $20,962,750,410 [41]. The numerator was the total dollar amount awarded to R-series grants for the FY of 2022 (*n* = 90). **Results:** For the FY 2022, $52,764,063 dollars were granted to 90 awards that focused on racism in healthcare (see Table 6). Of the 27 different NIH institutes/centers [36], 19 were funding agencies for on the 77 grants. The top four agencies/centers that allocated the most funds were— NIMHD (16.8%), NICHD (12.2%), NIDA (10%) and the NIMH (8.9%)—accounted for almost half (47.9%) of the funding for FY 2022. The NIH dollar amount funded for racism in health care during FY 2022 resulted in 0.25% ($52,764,063/$20,962,750,410). **Conclusion:** The results of this sub-analysis show that a small percentage of NIH monies is allocated towards racism in health care, even though we know important steps are needed to address racism. More funding of interventions and resources that address racism and it relationship to health disparities and inequities are needed. One of the study’s limitations is its dependence on the NIH’s RCDCs for themes linked to racism, which could reduce the amounts of grants connected to racism that result during a search.

**Table 6:** Case study: NIH’s funding agencies/centers, and funding dollar amounts focused on racism in healthcare – FY 2022 (*n* = 90)

Funding NIH Agencies/Centers		
	Number of funding agencies/centers (%)	Funding dollar amounts
AHRQ	1 (1.1%)	$ 50,000
NICHD ^a^	11 (12.2%)	$ 5,539,171
NCI	7 (7.8%)	$ 4,336,797
NHLBI	4 (4.4%)	$ 1,370,748
NIAID	1 (1.1%)	$ 721,171
NIAMS	1 (1.1%)	$ 791,024
NIDCR	2 (2.2%)	$ 707,974
NIDDK	1 (1.1%)	$ 756,925
NIEHS	3 (3.3%)	$ 956,502
NIGMS	1 (1.1%)	$ 552,246
NIMH	8 (8.9%)	$ 3,580,079
NINDS	1 (1.1%)	$ 100,000
NINR ^a^	7 (7.8%)	$ 4,829,559
NIA	7 (7.8%)	$ 5,339,615
NIAAA	1 (1.1%)	$ 727,945
NIDCD	1 (1.1%)	$ 717,005
NIDA	9 (10%)	$ 5,919,383
NIMHD	15 (16.8%)	$ 8,449,447
OD ^a^	9 (10%)	$ 4,318,472
	90 (100%)	$ 52,764,063
Funding dollar amounts: NIH Research	Projects on racism in healthcare	Overall research projects
	(*n* = 77)	(*n* = 38,426)
	$52,764,063 (0.25%)	$20,962,750,410

### Population focus

Nearly half (47.3%) of those studied in the 93 projects were racial and ethnic minorities (e.g., Black, Hispanic, Asian, American Indian/Alaska Native, Native Hawaiian/Pacific Islander) (Table 3). Most of the abstracts and/or public statements reported race or ethnicity (60.2%), and the largest racial and ethnic minority group studied were African American/Black (20.4%) individuals. Close to a quarter (24.7%) of the grants focused on more than one racial ethnic minority population and of these, 11.8% included both African American/Black and Hispanic/Latino/Latinx people in their studies. Of the 93 grants, most were aimed solely on patients (64.5%), followed by those on patients and providers (14%), on patients and policy (e.g., healthcare system; 8.6%), only on providers (6.5%), on the general population (3.2%) and another group that included students (3.2%; Table 3).

### Publications

Overall, from these 93 grants, there were a total of 278 publications produced; and 93% of them were from researchers funded by R01s, and 83.5% were from PAs or PARs. The majority of the publications were from multi-year grants that had grant lengths of 5-years or more (64.4%) and focused on system-wide racism (57.9%); while the remaining publications were of grants with a grant length of 4-years or less (33.8%) and on racism at the individual level (29.5%), or centered on both types of racism (system-wide and individual-level; 12.6%).

## Discussion

In most recent years, the recognition and acknowledgement of racism’s long history in American culture and modern medicine has shown the high economic toll it has, the unequal distribution of resources it creates and the increased health-risks and heath conditions that result from it, particularly among people of color and the Black populations [42]. In addition to being acknowledged as a public health crisis, racism has been shown to have a structural basis and to be deeply embedded in social programs and policies, which is known as structural racism [42, 43].

Different types of system-wide racism are considered upstream factors that influence health and are supported by discriminatory laws and policies, as they exacerbate already existing inequities [23, 42]. For example, a study showed structural and systemic racism as a major cause of maternal and infant health disparities; even after controlling for education and income, ethnic disparities in maternal and infant health persist, primarily due to differences in healthcare insurance coverage and access to care [29]. Confronting racism system-wide requires changing and dismantling the policies of institutions that support this form of discrimination, but also the culture of these systems [44]. Even though, measuring the impact of upstream factors may be difficult since it can take a substantial amount of time (e.g., a year or longer) to see the results [45, 46], the findings in this study showed NIH’s investment in studying structural racism, at almost 70% were on this type of racism. As identified by Hostetter & Klein [47], strategies to address system-wide racism include, using an upstream approach for hospitals and clinics to recognize ways racism affects their patients; offer training courses to providers and staff on implicit bias and antiracism principles; examine institutional policies with an antiracism and equity lens; review clinical algorithms that erroneously rely on race and limit treatment; create anonymous reporting initiatives to track and address racist behavior; and develop and implement guidelines on ways to address racist or prejudicial behaviors [47].

Racism at the system-level is equally as important as at the individual level, as they have a bi-directional relation to each other, and important upstream factors. For example, individual-level racism influences and reinforces broader systemic or structural patterns of oppression and vice versa [48]. Individual-level racism has been identified in the literature as implicit or explicit bias, which leads to discrimination against populations. Although explicit bias has declined over time, implicit bias remains [49]. Implicit bias permeates the healthcare system and affects patients in different ways, such as patient-clinician communication and rapport, trust towards providers, and/or clinical-decision making [49]. Implicit bias behaviors exhibited by healthcare providers towards their patients may partially explain health disparities among populations.

Approximately 40% of the grants did not mention the race and/or ethnicity of the population in the abstract or public statements, but the remaining studies did. Although the African American/Black population was the most studied, few grants solely focused on Asian individuals and no studies were only on Hispanic, American Indian, or Alaska Native and Pacific Islander groups. The lack of studies among these populations may create a gap in different types of research, such as applied (e.g., interventions that are culturally and linguistically appropriate), educational or population-based health disparities research.

Examples in the literature illustrate the need to study these populations. For instance, a study on Hispanic participants reported that 30% of them said healthcare providers lacked giving them the most advanced medical care; 31% were rushed by their healthcare provider during the medical visit; and 52% of those Hispanic individuals who identified as Black said, they had to speak up to get proper care from doctors compared to 31% of the Hispanic people who identified as White or 32% of those that identified as some other race [50]. Another study showed that non-Black oncologists who measured high in implicit racial bias had shorter interactions with their Black patients, resulting in less patient confidence in the recommended treatment plan [51]. Although there are no known effective evidence-based guideline to eliminate implicit bias, there are suggested ways individual providers acknowledge, identify and reduce implicit bias that include: introspection (e.g., self-reflection tools); use of different techniques (e.g., role playing and emotional regulation); and participation in bias and culturally competency trainings [52].

An aspect of racism that has been largely overlooked is internalized racism [53]. A study reported that symptoms of anxiety and discomfort were more common among Black participants who had higher degrees of internalized racism than among those who had lower levels [19]. Another study showed internalized racism to be linked to psychological distress (e.g., depression and anxiety) [16]. A review of the literature on internalized racism reported a need for more research on the experiences of different racial and ethnic groups, and on ways internalized racism intersects with other forms of internalized oppression [53]. The findings of this NIH portfolio analysis supports the literature, as it identified a gap in research since there were no NIH funded studies specifically addressing internalized racism, which is a concern as this type of racism continues to be ignored.

Financially, this portfolio analysis shows that from the FYs of 2017 to 2022, monies awarded towards racism in healthcare represent 0.07% of all NIH funding. Also, the 2022 FY case study reports that the investment for racism in healthcare was 0.25%. As NIH plays a pivotal role in supporting research that benefits the nation’s health and is committed to ending structural racism, the need to increase funding for opportunities such as internalized racism is essential [35]. Furthermore, racism is not one of the 315 RCDC spending categories created to conduct financial stewardship of NIH funding of all determinants [54, 55]. By it not being one of the categories, the amount of monies actually awarded on a yearly basis may be more and potentially are not fully captured.

## Limitations

There are several limitations of this portfolio analysis. One is based on the utilization of the NIH RePORT and reviewing the abstract and public health relevance statements which are brief, limiting the amount of available information on the grant. Another limitation is due to the lack of uniformity in the abstracts; and the lack of the use of the term “racism” in the abstract. This made it difficult to fully identify if the study was examining “racism,” possibly resulting in an under-inclusion and -estimation of racism in healthcare, despite the inclusion of grants that used terms or examples such as “antiracism lens” or “(e.g., structural racism).” A third limitation is related to the publications for each funded study. Because the purpose of this portfolio analysis was not to conduct any type of analysis on the content in the abstract, public health statements or study findings, the findings on publications do not go beyond mentioning the total number per grant and in its totality. Publications were reported to show the number of scientific advances that resulted from the grants. A fourth limitation is based on the term racism and it not being included as one of the 315 RCDC categories of spending. By it not being included as one of the terms, studies may not be categorized under racism resulting in an under-estimation of R-series grants on racism. A fifth limitation of this study is that the findings may not be generalizable to other countries as only studies conducted by US-based entities and within the US territories were included in this study. Lastly, by only including R-series grants in this review, relevant information could have been unintentionally excluded, impacting the results of this review.

## Conclusion

Findings from this portfolio analysis show that NIH has assigned monies toward studying racism in the healthcare settings, but also revealed the need to conduct more research on certain types of racism (i.e., internalized racism) and among all populations. This portfolio analysis also showed opportunities for NIH to: 1) request that investigators include in the abstract or public statement the population of focus; 2) the form of racism studied; and 3) broaden the RCDC spending categories to include “racism.”

As recognized by Dr. Perez-Stable, Director of NIMHD, structural racism is at the heart of many health disparities and inequities because it continues the long-standing social and health injustices [56]. For this reason, the CDC [55] recognizes that research on racism is needed and the focus needs to be farther upstream. These upstream factors, such as external determinants of health (governance and policies), need attention in order to better explain ways these determinants of health set in motion a progression of steps that many times result in health disparities [46]. Further research investigating ways these upstream factors create and impose downstream barriers, and that impact and play a role in determining these outcomes is vital [46].

## Data Availability

The data generated and analyzed in the current study are available in the NIH RePORTER (Research Portfolio Online Reporting Tools: Expenditures and Results) repository at https://reporter.nih.gov/.

## Abbreviations

*AHRQ*: Agency for Healthcare Research and Quality
*AN*: Alaska Native
*ADRDs*: Alzheimer ‘s Disease Related Dementias
*AIs*: American Indians
*BIPOC*: Black, Indigenous and peoples of color
*NICHD*: Eunice Kennedy Shriver National Institute of Child Health & Human Development
*FY*: fiscal year
*IR*: interpersonal racism
*MSM*: men that have sex with men
*NCI*: National Cancer Institute
*NHLBI*: National Heart, Lung, and Blood Institute
*NIAID*: National Institute of Allergy and Infectious Diseases
*NIAMS*: National Institute of Arthritis and Musculoskeletal and Skin Diseases
*NIDCR*: National Institute of Dental & Craniofacial Research
*NIDDK*: National Institute of Diabetes and Digestive and Kidney Diseases
*NIEHS*: National Institute of Environmental Health Sciences
*NIGMS*: National Institute of General Medical Sciences
*NIMH*: National Institute of Mental Health
*NIH*: National Institutes of Health
*NINDS*: National Institute of Neurological Disorders and Stroke
*NINR*: National Institute of Nursing Research
*NIA*: National Institute on Aging
*NIAAA*: National Institute on Alcohol Abuse and Alcoholism
*NIDCD*: National Institute on Deafness and other Communication Disorders
*NIDA*: National Institute on Drug Abuse
*OD*: NIH Office of the Director
*NIMHD*: National Institute on Minority Health and Health Disparities
*NHBs*: non-Hispanic Blacks
*NHWs*: non-Hispanic Whites
*NR*: Not reported
*PA*: *program announcement*
*PAR*: *program announcement receipt*
*PT*: patient
*POP*: population
*RCDC*: Research, Condition, and Disease Categorization
*RFA*: *requests for applications*
*SMS*: sexually minority men
*SR*: structural racism
*SYR*: systemic racism
*YBLMT*: Young Black or Latino men who have sex with men & transgender women
